# John Putnam Merrill: the unsung Hero behind the first successful renal transplantation

**DOI:** 10.3389/frtra.2025.1620534

**Published:** 2025-06-16

**Authors:** Reza Abdi

**Affiliations:** Brigham and Women’s Hospital, Harvard Medical School, Boston, MA, United States

**Keywords:** kidney, transplantation—kidney, transplantation, Merrill, Peter Bent Brigham Hospital

## Abstract

The first successful renal transplantation, performed between the Herrick twins at Peter Bent Brigham Hospital (PBBH) in 1954, was the culmination of four decades of collaboration among an exceptional group of physician-scientists and hospital leadership. PBBH was built with the primary goal of establishing an institution dedicated to biomedical research. John Merrill, a cardiologist who would go on to lay the foundation of modern nephrology, was perhaps one of the most important leaders in this effort. In addition to his pioneering transplantation work, Merrill developed what became the first functional dialysis machine that played a crucial role in stabilizing the diseased Herrick twin brother in preparation for the transplantation surgery—an operation that would go down in history as the world's first successful organ transplantation. Through these collective efforts, PBBH became the only hospital in the world at the time capable of offering a lifesaving dialysis-transplant procedure that combined both groundbreaking interventions. The dialysis-to-transplant model developed by Merrill has subsequently been adopted worldwide and saved thousands of lives.

## Introduction

Perhaps it is safe to say that organ transplantation has captivated human imagination more than any other medical intervention throughout history. This dream journeyed from ancient civilizations such as Mesopotamia, Egypt, and Persia, through later civilizations such as ancient Rome, and eventually to modern Western civilizations (1). However, the true realization of this dream occurred with the first successful renal transplantation in 1954 at Peter Bent Brigham Hospital (PBBH), a modest hospital established 40 years earlier in a poor Boston neighborhood. The hospital was built with funds provided by Peter Bent Brigham to support “the care of sick persons in indigent circumstances” (2, 3). Like any monumental achievement, the success of organ transplantation ultimately boiled down to one word: *leadership*.

The collaborative efforts that culminated in this historic breakthrough could arguably be considered to have begun four decades earlier, with the decision to found PBBH with an eye towards biomedical research. Around the turn of the century, there was a growing ambition to build hospitals focused on biomedical research rather than standard clinical practice. Harvard President Charles Eliot, inspired by the Johns Hopkins model, sought to establish such an institution. With Massachusetts General Hospital already a major clinical center, Eliot pursued a very ambitious venture, aimed at building a hospital in close physical proximity to Harvard Medical School. Fortuitously, funds set aside by Peter Bent Brigham after his death some 25 years earlier had matured, enabling Eliot to collaborate with the Brigham Corporation to acquire the necessary land and build PBBH. The affiliation of Harvard Medical School with PBBH was soon followed by Harvard's affiliation with other institutions, including Boston Children's Hospital. This unprecedented expansion of biomedical research institutions created a hub of scientific advancement unlike anything seen before, laying the foundation for groundbreaking medical innovations, including the historic achievement of organ transplantation.

**Figure 1:**
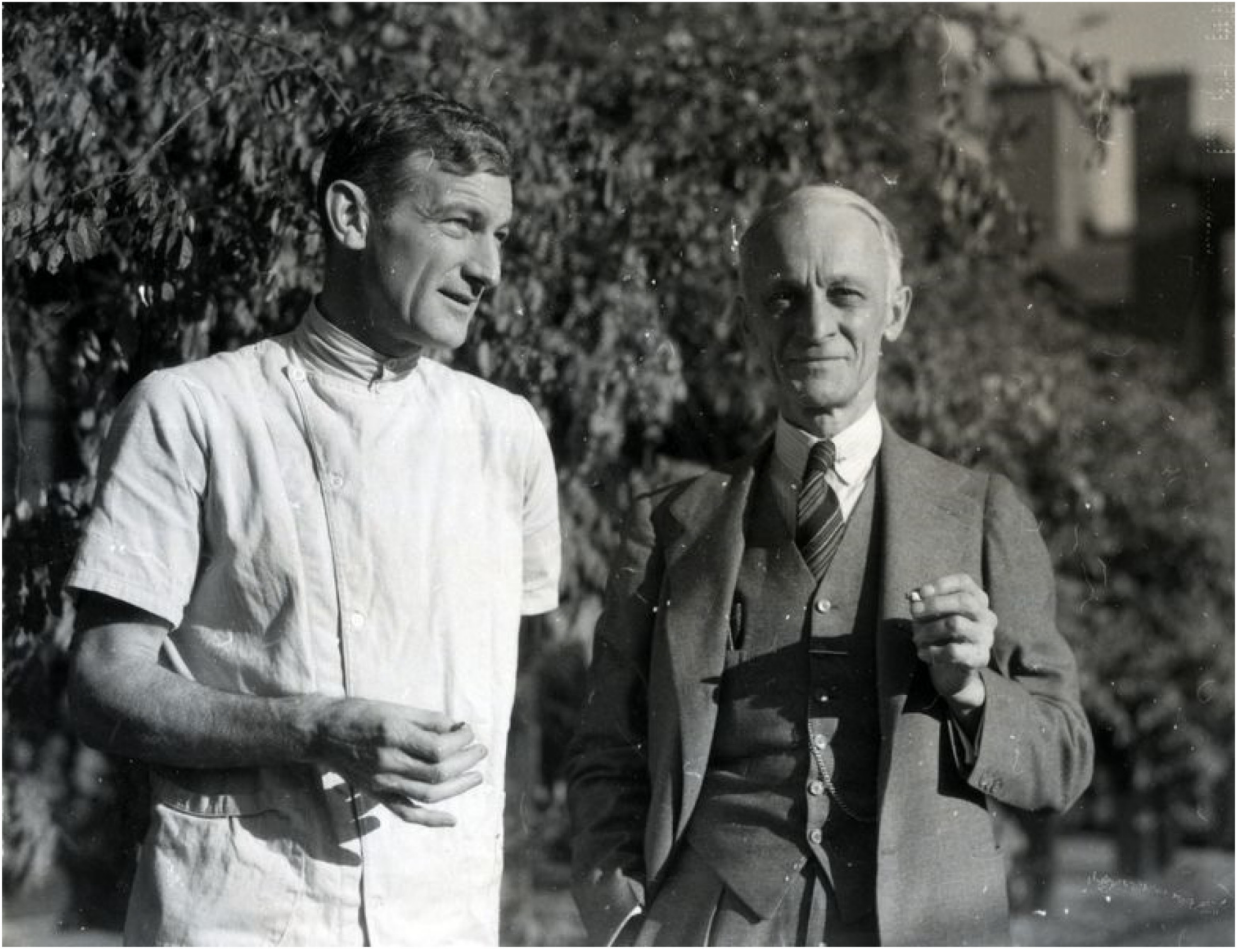
Elliot Cutler (left) and Harvey Cushing (right). Source: Brigham and Women's Hospital Archives.

With this visionary affiliation and surroundings, PBBH set out to attract the most distinguished minds in biomedical research, establishing itself as a center for pioneering medical discoveries and innovation. One key recruit was Harvey Cushing (Figure 1), who would go on to become arguably the most influential surgeon in the history of medicine. He brought his protégé William Quinby, a urologist who pioneered the autotransplantation of kidneys in dogs in 1915. Quinby trained J. Hartwell Harrison, who later performed the high-risk kidney removal from the donor twin, probably riskier than Joseph Murray's transplant procedure on the diseased twin (4).

**Figure 2:**
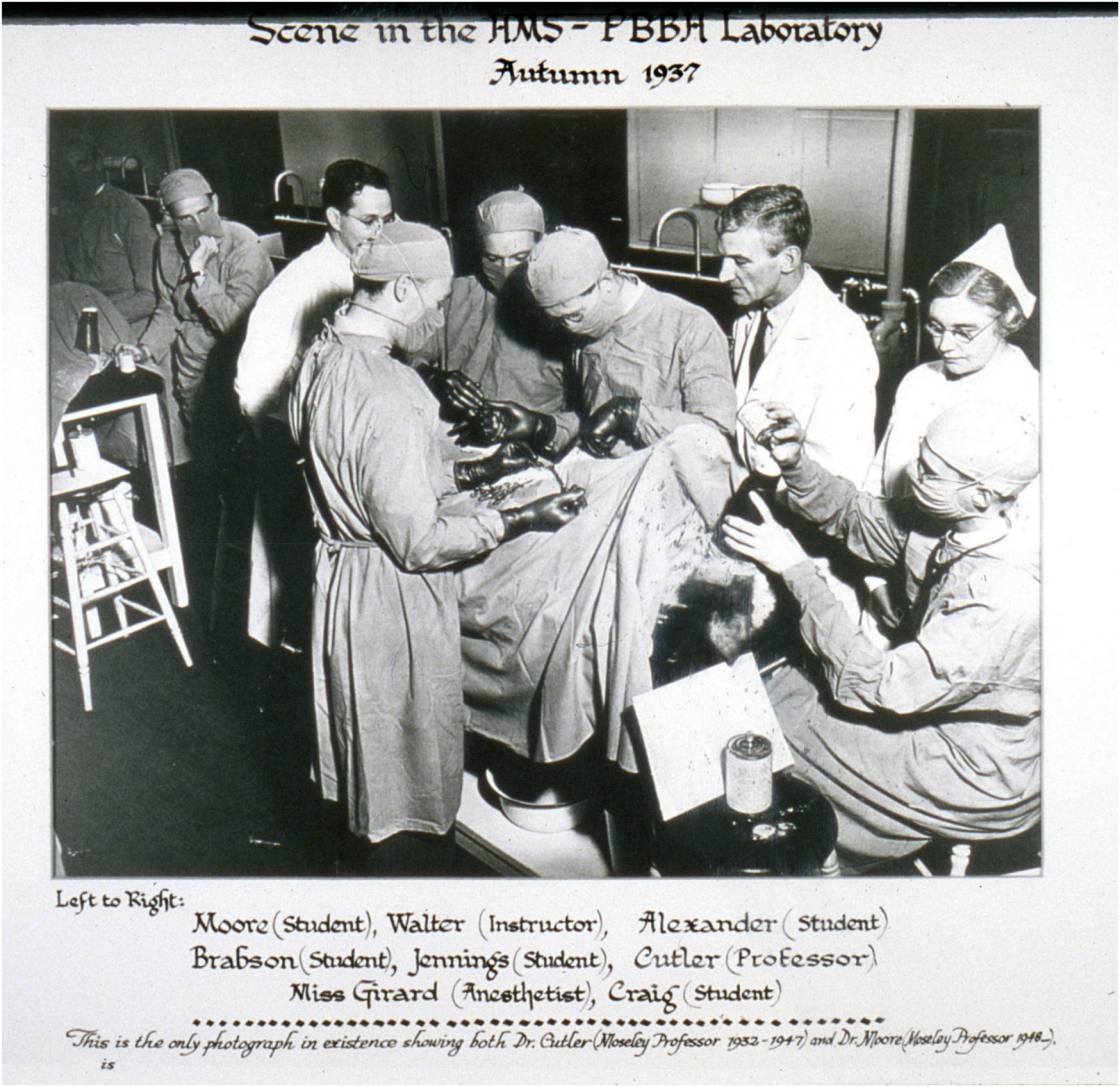
Experimental surgery in canine models. Source: Brigham and Women's Hospital Archives.

At its inception, PBBH faced skepticism and financial challenges so significant that many doubted whether the hospital would survive beyond its first few years. Some of the assets left behind by Peter Bent were not effectively managed, exacerbating the hospital's financial difficulties. The situation worsened following the death of Elliot Cutler, plunging the institution into a significant crisis. In 1923, nearly a decade after the opening of PBBH, the Chief of Surgery, Elliot Cutler (Figures 1, 2), in collaboration with his cardiology colleague Samuel Levine, performed the first closed transventricular mitral commissurotomy on a patient dying of rheumatic mitral stenosis. Despite these struggles, the hospital's leadership made a bold decision—not to transform it into a profit-driven institution but to remain committed to its mission of advancing biomedical research. In a high-risk move, they appointed Dr. Francis Moore (Figure 2), a surgeon with extensive research experience, as Surgeon-in-Chief, reinforcing their dedication to scientific progress. At 34, Moore became the youngest Chairman of Surgery in Harvard's history and served as the Moseley Professor of Surgery at HMS. Under his leadership, the Department of Surgery became a pioneering center for surgical research and innovation, particularly in advancing transplant programs.

On the internal medicine front, George Thorn transformed the Department of Medicine at PBBH from a department with few house staff into perhaps the best department of medicine in the world. George Thorn had extensive research experience in the roles of the adrenal glands and kidneys in blood pressure regulation. Though often unrecognized, he played a crucial role in orchestrating the hospital's transplant program. Notably, Thorn was behind an earlier, lesser-known kidney transplant case that took place a few years before Joseph Murray's landmark successful transplant. This earlier procedure was conducted in a patient's room under the light of a gooseneck lamp. However, the case was not widely discussed, possibly due to concerns about the surrounding controversy. While the patient did recover, it remained unclear whether the kidney transplant had contributed to the recovery.

Another of Thorn's crucial roles was his decision to appoint John Putnam Merrill, a cardiologist at the time, to lead the new dialysis program. Finally, George Thorn's contribution to medical research nationwide cannot be underestimated, as his connection with Howard Hughes led to the establishment of the Howard Hughes Medical Institute (HHMI). The HHMI has been one of the most significant single-donor philanthropic contributors to scientific research, supporting groundbreaking discoveries and funding more than 30 Nobel laureates. John Merrill and his dialysis work were among the early recipients of HHMI funding. Ironically, Howard Hughes himself died of renal failure without dialysis access, despite his institute's profound impact on advancing kidney treatment and medical research.

**Figure 3:**
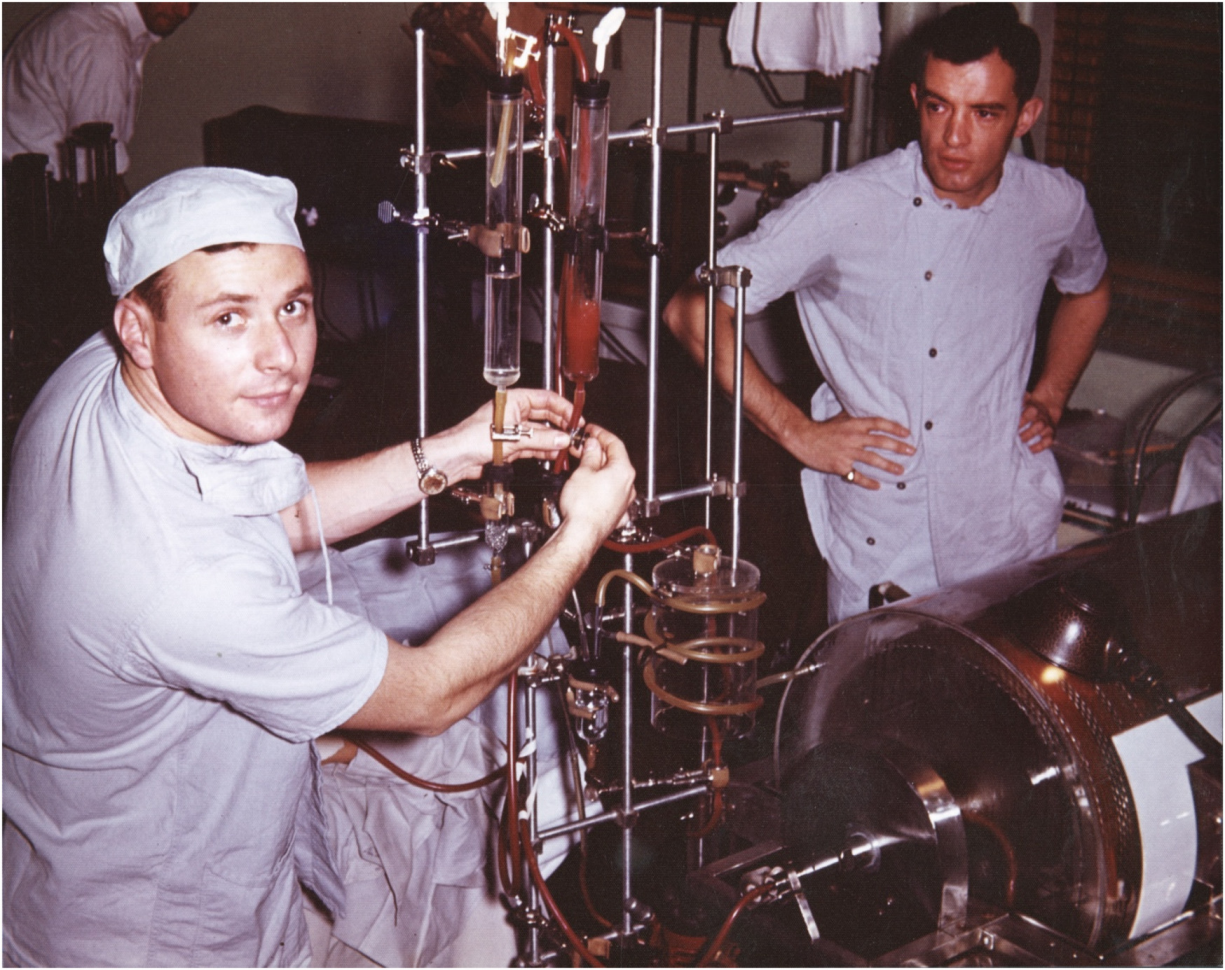
Chester Rosoff **(L)**, John P. Merrill **(R)**, and artificial kidney machine, circa 1948. Source: Carl W. Walter papers, HMS c150, Harvard Medical Library collection, Center for the History of Medicine in the Francis A. Countway Library, Harvard University.

**Figure 4:**
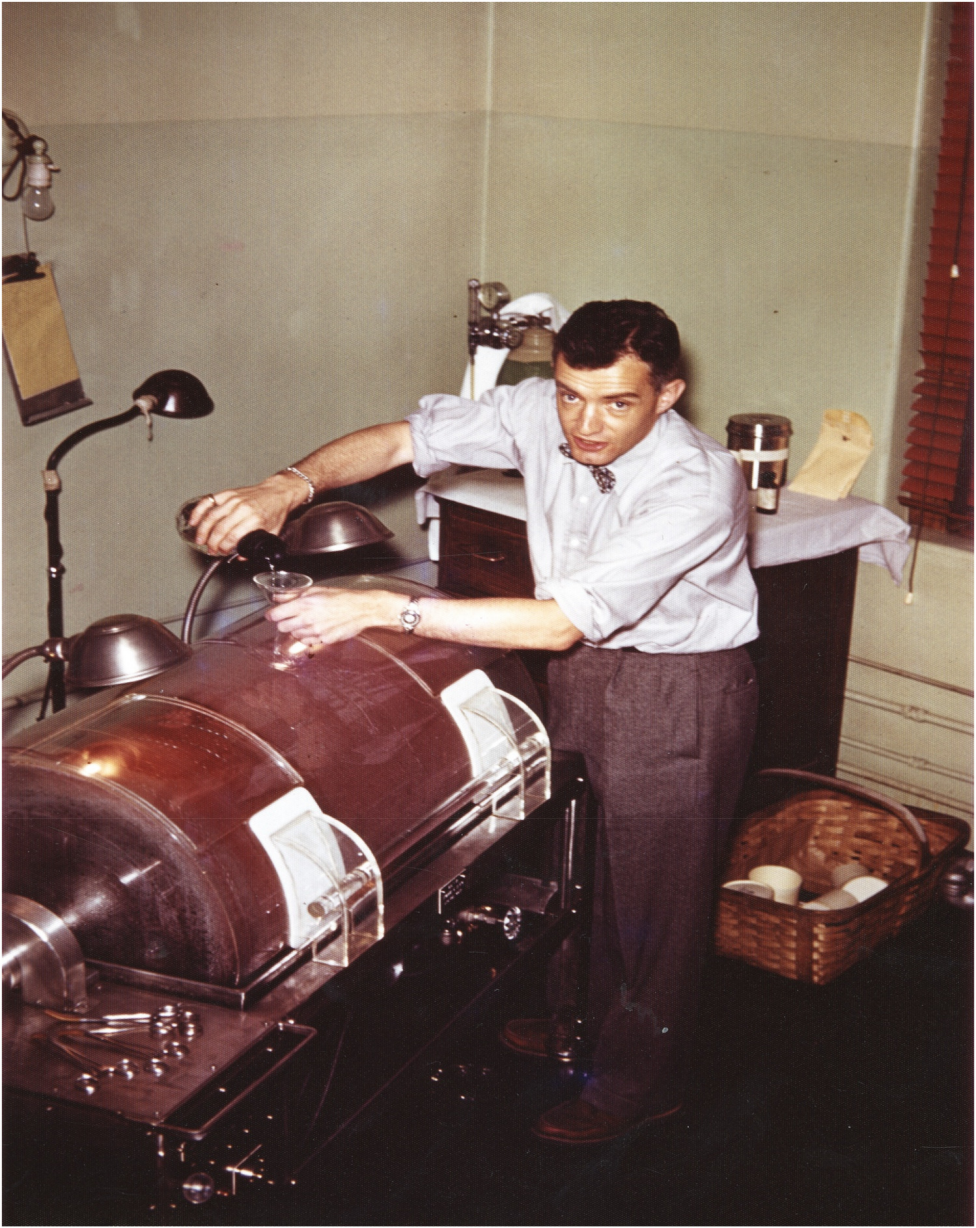
John P. Merrill working on his artificial kidney prototype, circa 1948. Source: Carl W. Walter papers, HMS c150, Harvard Medical Library collection, Center for the History of Medicine in the Francis A. Countway Library, Harvard University.

When Willem Kolff built the first dialysis machine in 1945, he sent several prototypes to different programs, but they were poorly received. One of his prototypes was sent to a major academic program in England, but it was reportedly dismantled and repurposed for plumbing—an indication that the medical world was not yet ready for such interventions. Although Kolff had no prototype available to offer to PBBH, he was happy to accept a speaking invitation from George Thorn. Although Kolff did not meet him directly at the time, Merrill was in the audience. Thorn and Merrill later brought in Carl Walter, a surgeon, who further consulted with Edward Olsen, an engineer. When Olsen examined Kolff's blueprints, he confidently stated that he could build a better prototype. Merrill worked tirelessly with a team to improve Kolff's largely non-functional dialysis machine, including the purchase of cellophane casings from Viscase Casings, a company that sold sausage casings. Finally, with the help of Olsen, Merrill built the first functional but clumsy prototype in his garage. Among the many individuals who supported the development of hemodialysis at PBBH was Dr. Murphy, the son of the first Nobel Laureate at PBBH, Dr. William P. Murphy, Sr., who was honored for his pioneering work on the treatment of pernicious anemia, leading to the discovery of Vitamin B12. Interestingly, Merrill's initial interest in dialysis was to study the impact of dialysis on electrolytes and their effects on the heart; this work led him to be the first to describe the scientific basis for potassium toxicity and its associated EKG changes. Only later did Merrill's focus change to the potential for dialysis as a life-saving intervention for patients in renal failure. Merrill's subsequent dialysis initiatives, though mocked in their early years, eventually saved millions of lives (5, 6) (Figures 3, 4).

Most historical accounts on the first successful renal transplant of the Herrick twins, written virtually all by surgeons, do not appreciate how ill Richard Herrick was. As Merrill put it, the patient “had all the stigmata of malignant hypertension.” More specifically, Richard exhibited evidence of heart failure and retinal hemorrhage, was clinically unstable with respect to volume, suffered from severe hypertension, and had seizures alongside advanced uremic encephalopathy. Patients in this condition, even with today's advancements, would likely not move forward with surgery. However, Merrill was able to dialyze Richard with the machine he had refined over the years, stabilizing him sufficiently to undergo surgery. This dialysis-to-transplant model, originally developed by Merrill and Hume, is currently used worldwide and has saved tens of thousands of lives.

It is important to note that 4 years prior to the first successful transplantation, the transplant and hemodialysis procedures had been initiated and steadily optimized at PBBH by John Merrill. During this period, surgeon David Hume—a highly skilled and pioneering figure in surgical innovation who sought to follow in the footsteps of his predecessor, Harvey Cushing—played a crucial role at PBBH. Hume was renowned for his meticulous technique and for developing surgical methods essential to vascular and organ transplantation. At PBBH, he led early experimental kidney transplants in dogs and was instrumental in establishing the surgical protocols that would later enable the first successful human kidney transplantation. His expertise and innovative approaches laid critical groundwork for the field's advancement.

Joseph Murray, a plastic surgeon who had been introduced to the world of transplantation by Hume, began training in kidney transplantation in dogs after Hume left PBBH to serve in the Korean War. At that time, Hume had developed a technique in which the kidney was placed in the anterior thigh, with the ureter brought out through the skin for urinary drainage. Donor kidneys were obtained, in collaboration with Chief of Pediatric Neurosurgery Donald Matson, from Children's Hospital, where patients with hydrocephalus had one kidney removed to accommodate a shunt (7, 8). Without immunosuppressants, all nine grafts were rejected, except for one that lasted months due to advanced uremia-induced immunosuppression. This case inspired the team to pursue interventional means of immunosuppression in later work.

**Figure 5:**
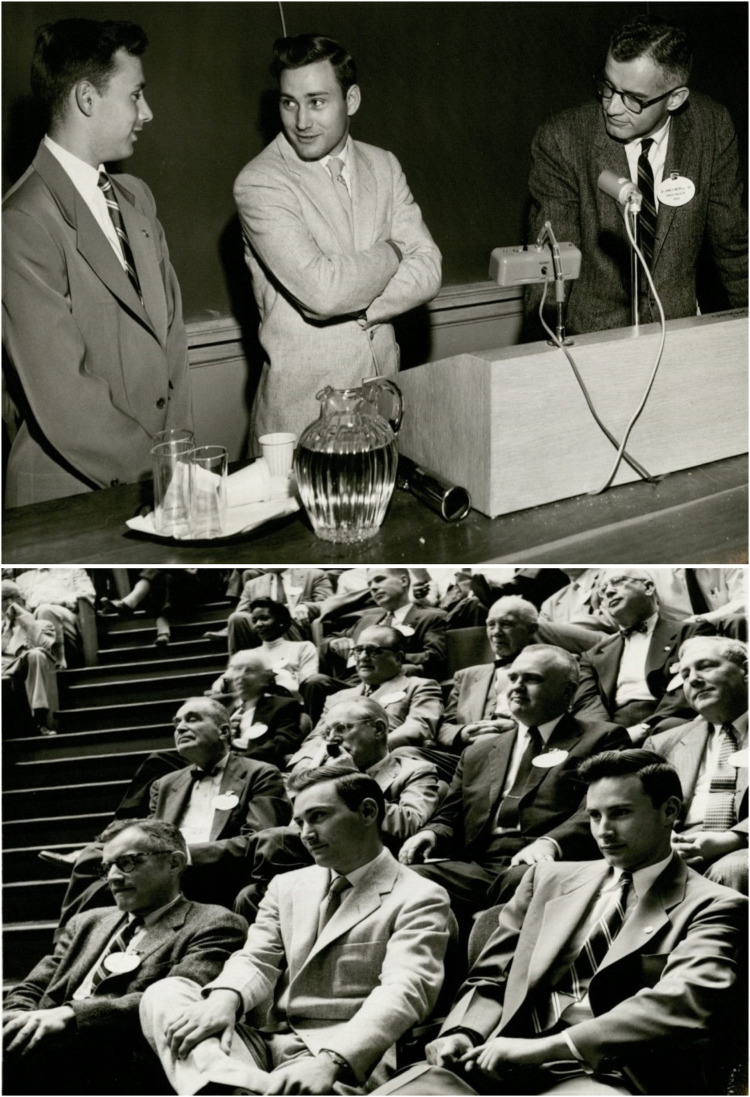
Merrill with the twin brothers. Source: Center for the History of Medicine in the Francis A. Countway Library, Harvard University.

An astonishing aspect of the story was Dr. David C. Miller of the Public Health Service Hospital in Boston, who offered hope to the Herrick family by referring both twins to Merrill for dialysis and transplantation (Figure 5). This may be attributed to the dissemination of the extensive research activities at the PBBH, led by Merrill and others, to nearby centers and primary care physicians. Once the brothers had been referred, Merrill's team employed numerous methods, from checking eye color to fingerprinting at a police station in West Roxbury, to confirm the twin brothers were indeed identical twins. They then proceeded to test skin transplants across both brothers to provide the ultimate proof of immunocompatability (9).

Tackling the immune rejection amongst nonidentical twins, the Brigham transplant group also tested irradiation as immunosuppression method in humans between 1958 and 1962. James Dealy, Radiologist-in-Chief, oversaw the first total body irradiation (TBI). The grim results of this initial effort led to a shift to sublethal irradiation. The development of sublethal radiation procedures was greatly enabled by Merrill, who had gained experience in radiation exposure by participating as a physician in “Operation Crossroads,” a project replicating the Hiroshima bomb. With the help of the pathologist Warren Shields, Merrill's team successfully estimated the sublethal irradiation dose for transplant patients, leading to the first successful long-term transplant among non-identical twins. The only successful use of TBI at the Brigham occurred in 1959 with fraternal twins, John and Andrew Riteris. John Riteris lived for many decades with a transplanted kidney.

**Figure 6:**
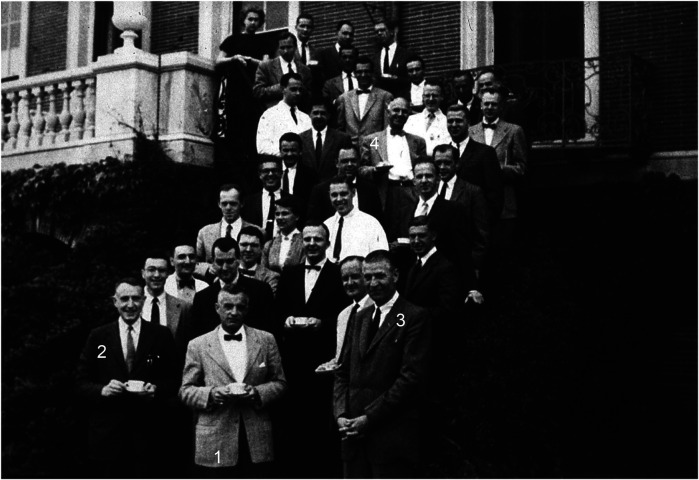
From front to back, the figure shows (1) John Merrill, (2) Francis Moore, (3) Gustave Dammin (pathologist-in-chief), and (4) Joseph Murray. Source: Brigham and Women's Hospital Archives*.*

With the development of dialysis and the success of the first renal transplant at PBBH, the institution quickly became a global hub—the only program in the world capable of offering both treatments. As a result, patients began traveling to Boston to receive care. This nationwide attraction helped propagate the PBBH program. John Merrill became the face of this success, rising to national prominence and even achieving celebrity status. Following the first successful identical twin transplant, several other twin transplants were performed, including one involving a donor from Sweden. As these cases progressed, some patients developed glomerulonephritis (GN), leading Merrill to lay the foundation for a new line of investigation in the field aimed at understanding recurrent GN in transplanted kidneys. Major figures behind the first successful kidney transplantation at PBBH are depicted in Figure 6. As Thomas Starzl put it, “The hospital's ruling board and administrative structure did not falter in their support of the quixotic objective of treating end-stage renal disease.”

On (10) the other side of the Atlantic, the French have played a dominant role in transplantation from the very beginning, pioneering advancements such as xenotransplantation in humans, the first human renal transplants, and innovative techniques for implanting organs in the groin. Merrill had a close friendship with Jean Hamburger at Necker Hospital in Paris, one of the founding fathers of modern nephrology. In 1952, Merrill traveled to Paris to support Hamburger during the first living kidney donation, although the young recipient tragically died due to graft rejection. Their correspondence often pushed the boundaries of medical experimentation, with Merrill encouraging Hamburger to continue his efforts.

Merrill authored hundreds of scientific articles and the seminal textbook, *The Treatment of Renal Failure*, over his career. Born in Hartford, Connecticut, on March 10, 1917, Merrill graduated from HMS in 1942 and subsequently served as an army physician for 4 years. There his experience in Operation Crossroads experience profoundly impacted his life, fueling his desire to combat weapons of mass destruction. Once he returned to civilian practice, he was first supported by a Mellon Fellowship (Harvard, 1949), served as an investigator for the American Heart Association from 1950 to 1957, and became the Director of the Cardio-Renal Service at PBBH in 1952, a time when the term “nephrology” had not yet been coined. His accomplishments were recognized through a position as an investigator for the HHMI, a role he held between 1957 and 1970. He received the Amory Prize from the American Academy of Arts and Sciences in 1962, and he was awarded the Gairdner Foundation Award, often been given to future Nobel laureates, in 1969.

**Figure 7:**
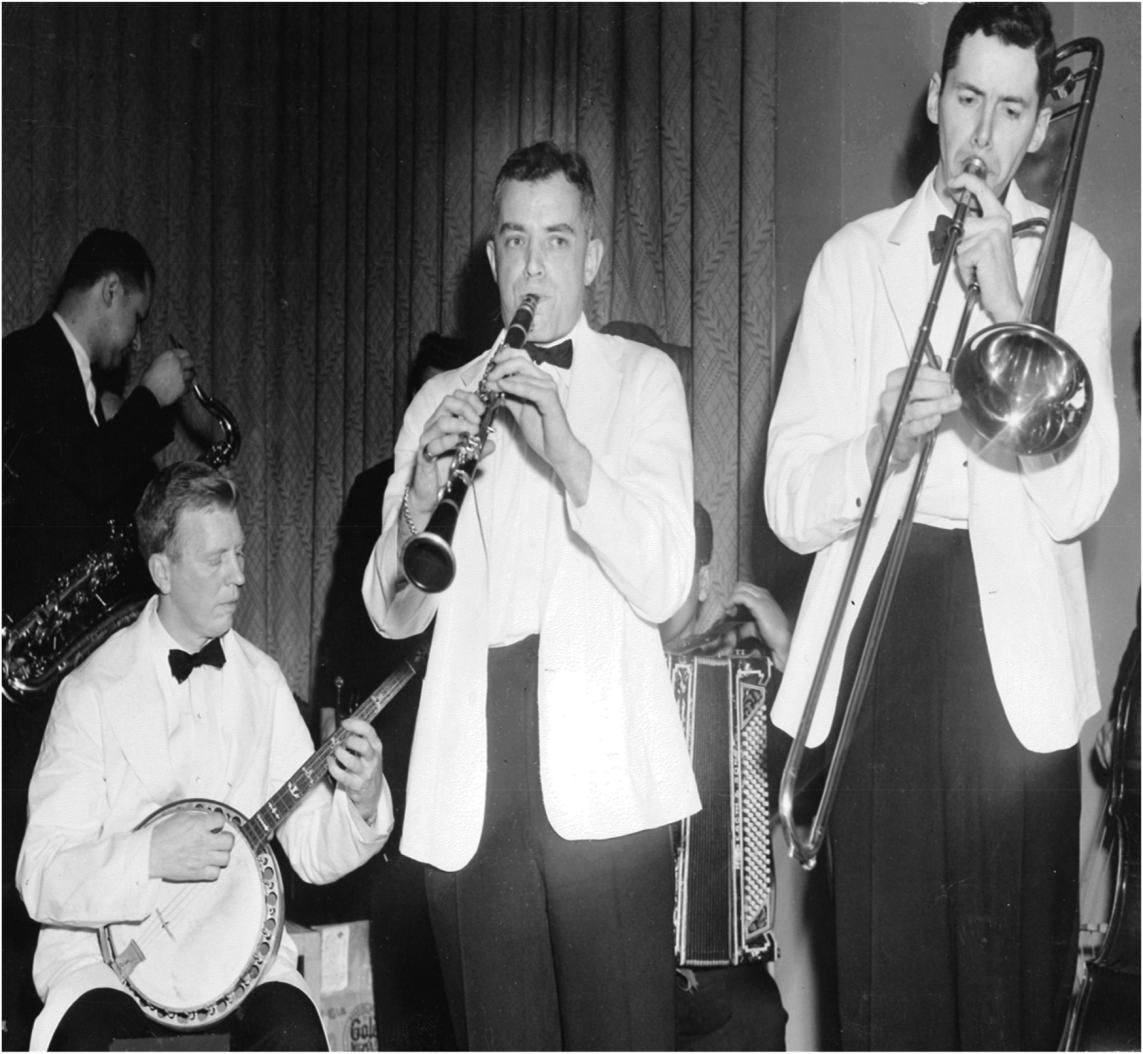
Dixieland jazz band. George Thorn on banjo (left) and John Merrill on clarinet (middle).

Merrill's interests spanned far beyond medicine. A talented musician, Merrill wrote musicals for PBBH staff and played the clarinet in a Dixieland jazz band called the “Malady Boys” with George Thorn (Figure 7). He was also an accomplished sailor, swimmer, and tennis player. Merrill's entourage included individuals who shared his love of Paris, Africa, and scuba diving—interests famously associated with Ernest Hemingway (11, 12).

Merrill passed away on April 4, 1984, in Hopetown, Bahamas, from drowning. His premature death may have prevented him from receiving even greater recognition, such as sharing the Nobel Prize with Murray, for his landmark contributions to the fields of transplantation and kidney disease.

## Data Availability

The original contributions presented in the study are included in the article/Supplementary Material, further inquiries can be directed to the corresponding author.
